# Clinical Assay for the Early Detection of Colorectal Cancer Using Mass Spectrometric Wheat Germ Agglutinin Multiple Reaction Monitoring

**DOI:** 10.3390/cancers13092190

**Published:** 2021-05-02

**Authors:** I-Jung Tsai, Emily Chia-Yu Su, I-Lin Tsai, Ching-Yu Lin

**Affiliations:** 1 Ph.D. Program in Medical Biotechnology, College of Medical Science and Technology, Taipei Medical University, Taipei 11031, Taiwan; d609108005@tmu.edu.tw; 2 Graduate Institute of Biomedical Informatics, College of Medical Science and Technology, Taipei Medical University, Taipei 11031, Taiwan; emilysu@tmu.edu.tw; 3 Clinical Big Data Research Center, Taipei Medical University Hospital, Taipei 11031, Taiwan; 4 Department of Biochemistry and Molecular Cell Biology, School of Medicine, College of Medicine, Taipei Medical University, Taipei 11031, Taiwan; isabel10@tmu.edu.tw; 5 Graduate Institute of Medical Sciences, College of Medicine, Taipei Medical University, Taipei 11031, Taiwan; 6 School of Medical Laboratory Science and Biotechnology, College of Medical Science and Technology, Taipei Medical University, Taipei 11031, Taiwan

**Keywords:** colorectal cancer, multiple reaction monitoring, tandem mass spectrometer, wheat germ agglutinin, plasma, machine learning algorithm

## Abstract

**Simple Summary:**

Colorectal cancer (CRC) is currently the third leading cause of cancer death worldwide. Early diagnosis of CRC is important for increasing the opportunity for treatment and receiving a good prognosis. The aim of our study was to develop a detection method that combined wheat germ agglutinin (WGA) chromatography with mass spectrometry (MS) for early detection of CRC. Further, machine learning algorithms and logistic regression were applied to combine multiple biomarkers we discovered. We validated in a population of 286 plasma samples the diagnostic performance of peptides corresponding to WGA-captured protein and its combination, which received a sensitivity of 84.5% and a specificity of 97.5% in the diagnoses of CRC. Proteomic biomarkers combined with algorithms can provide a powerful tool for discriminating patients with CRC and health controls (HCs). Measurements of WGA-captured PF4, ITIH4, and APOE with MS are then useful for early detection of CRC. Additionally, our study revealed the potential of applying lectin chromatography with MS for disease diagnosis.

**Abstract:**

Colorectal cancer (CRC) is currently the third leading cause of cancer-related mortality in the world. U.S. Food and Drug Administration-approved circulating tumor markers, including carcinoembryonic antigen, carbohydrate antigen (CA) 19-9 and CA125 were used as prognostic biomarkers of CRC that attributed to low sensitivity in diagnosis of CRC. Therefore, our purpose is to develop a novel strategy for novel clinical biomarkers for early CRC diagnosis. We used mass spectrometry (MS) methods such as nanoLC-MS/MS, targeted LC-MS/MS, and stable isotope-labeled multiple reaction monitoring (MRM) MS coupled to test machine learning algorithms and logistic regression to analyze plasma samples from patients with early-stage CRC, late-stage CRC, and healthy controls (HCs). On the basis of our methods, 356 peptides were identified, 6 differential expressed peptides were verified, and finally three peptides corresponding wheat germ agglutinin (WGA)-captured proteins were semi-quantitated in 286 plasma samples (80 HCs and 206 CRCs). The novel peptide biomarkers combination of PF4^54–62^, ITIH4^429–438^, and APOE^198–207^ achieved sensitivity 84.5%, specificity 97.5% and an AUC of 0.96 in CRC diagnosis. In conclusion, our study demonstrated that WGA-captured plasma PF4^54–62^, ITIH4^429–438^, and APOE^198–207^ levels in combination may serve as highly effective early diagnostic biomarkers for patients with CRC.

## 1. Introduction

Colorectal cancer (CRC) is currently the third leading cause of cancer-related mortality worldwide and the most common cancer in Taiwan [1,2]. The global incidence by the year 2030 is estimated to be 2.2 million new cases, with the number of CRC-related deaths for that period to be 1.1 million [3]. Currently, the guaiac fecal occult blood test (gFOBT) is the most common non-invasive screening method, which is based on detecting the activity of hemoglobin peroxidase. Despite the FOBT being a rapid and low-cost method for screening CRC, poor selectivity and sensitivity by FOBT result in high rates of false positives and false negatives [4]. Various circulating protein biomarkers, including carbohydrate antigen 125 (CA125), CA19-9, carcinoembryonic (CEA), alpha fetoprotein (AFP), and ferritin, are applied for monitoring and diagnosing CRC in the clinic; however, these biomarkers only achieved 10.39–46.59% sensitivity and 80~95% specificity in diagnosing CRC [5,6]. Therefore, an alternative rapid, high throughput and accurate screening procedure is urgently needed for early diagnosis of CRC.

Liquid chromatography coupled with tandem mass spectrometry (LC-MS/MS) has been widely used for proteomic studies [7]. For example, Saleem S. et al. revealed that caveolin-1 and matrix metalloproteinase-9 are stage dependent in CRC in proteomic analysis [8]. Furthermore, it has been applied for biomarker discovery in qualitative and quantitative analyses [9]. An elegant study from Shiromizu T. et al. identified and validated 22 biomarker candidates for CRC with LC-MS/MS [10]. Moreover, Beretov J. et al. identified biomarker candidates with label-free LC-MS/MS [11]. Label-free LC-MS/MS can provide global characterization of proteomic features to identify biomarker candidates; however, the findings required further verification and validation [9]. Therefore, verification and validation of findings from label-free LC-MS/MS usually use isotope-label multiple reaction monitoring (MRM) which provides more-sensitive and more-reliable quantitative results [12]. In an MRM-based assay, analyzed by LC-MS/MS, targeted transitions of precursors selected in Q1 and fragment ions selected in Q3 are monitored by a triple-quadrupole MS instrument that generates signals for qualification and quantitation [12]. MRM-based assays have been widely applied to protein quantitation in various fluids, including plasma [13] and serum [14].

Glycan can affect tumor progression in different ways, including metastasis, invasion, and proliferation [15,16]. The heterogeneity of glycosylation sites or changes in glycan structures in body fluids were shown to be correlated with the development and progression of certain cancer states [17,18]. Further, alteration of glycoprotein levels was reported in CRC [19], breast cancer [20], and prostate cancer [21]. Therefore, glycoproteins can be considered an ideal source for the early detection of cancers [22]. For instance, many cancer biomarkers in the clinic are glycoproteins, including CEA in CRC [6], carbohydrate antigen 19-9 (CA19-9) in gastrointestinal cancer [23], and prostate-specific antigen (PSA) in prostate cancer [24]. Lectin chromatography has been widely used to capture glycoproteins for MS analyses [25,26]. Lectins are a group of proteins that have unique affinities to carbohydrates; they can reversibly and specifically interact with certain glycan structural motifs [27]. For example, wheat germ agglutinin (WGA) can bind with N-acetylglucosamine (GlcNAc) on glycoproteins and interact with sialic acid-containing oligosaccharides [28]. Studies showed that the addition or removal of *O*-linked N-acetylglucosamines on proteins very likely plays key roles in tumor pathogenesis [29]. However, only a few studies have applied lectin affinity chromatography with LC-MS/MS in biomarker discovery and validation [30,31,32].

Hence, in this study we performed WGA chromatography and nanoLC-MS/MS to discover biomarker candidates in patients with CRC and utilized ultra-performance (UP) LC-MS/MS to perform a targeted method to verify our biomarker candidates. We then synthesized the verified biomarker candidates and optimized the LC-MS/MS parameters. To further examine the biomarker performance, a stable isotope-labeled MRM assay with machine learning algorithms was used to validate the performance of the diagnostic biomarkers. In short, after biomarker discovery and analytical method development, 80 plasma samples from healthy controls (HCs) and 206 plasma samples from patients with CRC were analyzed; a set of biomarkers consisting of platelet factor 4 (PF4^54–62^), apolipoprotein E (APOE^198–207^) and inter-alpha-trypsin inhibitor heavy chain H4 (ITIH4^429–438^) showed significant differences among HCs, early-stage CRC patients, and late-stage CRC patients. Moreover, machine learning algorithms and logistic regression were incorporated to combine the diagnostic performances of the biomarkers.

## 2. Materials and Methods

### 2.1. Materials

The synthetic peptides and their stable isotope-labeled counterparts were obtained from Yao-Hong Corp. (New Taipei City, Taiwan). Sequencing-grade trypsin was purchased from Promega (Madison, WI, USA). Chicken serum was purchased from MyBioSource (San Diego, CA, USA). Agarose-bound wheat germ agglutinin (WGA) was obtained from Vector Laboratories (Burlingame, CA, USA). N-Acetylglucoseamine and formic acid were purchased from Tokyo Chemical Industry (Tokyo, Japan). Acetonitrile, methanol, dithiothreitol (DTT), and iodoacetamide (IAM) were obtained from Sigma-Aldrich (St. Louis, MO, USA).

### 2.2. Patients and Specimens

Plasma samples from 286 patients with CRC and 120 healthy volunteers (healthy controls (HCs)) were all purchased from the Joint Biobank of Taipei Medical University. This study was approved by the Taipei Medical University-Joint Institutional Review Board (nos. 201308022 and N202007061). As for the sample collection procedure in biobank, the whole blood samples were withdrawn in EDTA tubes and centrifuged at 3500 rpm for 5 min. After centrifugation, the plasma samples were stored at −80 °C until analyzed. Information on patient demographics and clinical features are summarized in Table 1. CRC plasma samples were from patients with stage I, II, III, and IV tumors. The pooled samples of CRC were generated into CRC stage I, CRC stage II, CRC stage III, and CRC stage IV from 80 CRC plasma samples. As for the pooled samples of the healthy control, they were generated into HCs from 40 HC plasma samples. The following validation set of 286 samples (early-stage CRC (stage I/II tumors, 100 samples), late-stage CRC (stage III/IV tumors, 106 samples), and healthy controls (80 samples)) were sampled from independent plasma samples of CRC patients and HCs.

### 2.3. Wheat Germ Agglutinin Chromatograhpy and Sample Preparation

20 μL of plasma sample was added and mixed with agarose bound WGA for 1 h. WGA-bound proteins were washed with phosphate-buffered saline (PBS) three times and eluted with elution buffer (0.5 M N-acetylglucosamine dissolved in 1 mM acetic acid). The Bradford assay was conducted to determine protein concentrations after elution. Proteins (20 μg) were lyophilized in a SpeedVac system. Extended isotope-labeled peptides or BSA were spiked into the sample after the protein pellet had been reconstituted in 30 μL of double-distilled (dd) H_2_O. DTT (550 mM, 1 μL) was added to the sample and incubated at 56 °C for 45 min. After 2 μL of IAM (450 mM) was added to the sample and incubated in the dark for 45 min, the sample was digested with trypsin (0.5 μg) for 16 h at 37 °C. Digestion was quenched by adjusting the final concentration in 0.1% formic acid.

### 2.4. Nano-LC-MS/MS Analyses

The nano-LC-MS/MS analysis was performed on a nanoAcquity system (Waters, Milford, MA, USA) connected to an Orbitrap Elite hybrid mass spectrometer (Thermo Electron, Bremen, Germany) equipped with a PicoView nanospray interface (New Objective, Woburn, MA, USA). Peptide mixtures were loaded onto a 75-μm inner diameter, 25-cm-long C18 BEH column (Waters) packed with 1.7-μm particles with a pore width of 130 Å and were separated using a segmented gradient in 60 min from 5% to 35% solvent B (acetonitrile with 0.1% formic acid) at a flow rate of 300 nl/min and a column temperature of 35 °C. Solvent A was 0.1% formic acid in water. The mass spectrometer was operated in the data-dependent mode. Briefly, surveyed full-scan MS spectra were acquired in the orbitrap (m/z 350~1600) with resolution set to 120 K at m/z 400 and an automatic gain control (AGC) target of 10^6^. The 20 most intense ions were sequentially isolated for collision induced dissociation MS/MS fragmentation and detection in a linear ion trap (AGC target of 10^4^) with previously selected ions dynamically excluded for 60 s. Ions with a single and unrecognized charge state were also excluded. PEAKS 7 software (Bioinformatics Solutions, Waterloo, ON, Canada) was used to sequence WGA-captured proteins from acquired MS/MS spectra against the Universal Protein Knowledgebase, a human protein database (UniProt; http://www.uniprot.org/, 18 January 2020) containing 168,088 protein entities (UniProt, January 2020) with MS tolerance set to 10 ppm, MS/MS tolerance set to 0.6 Da, and with a false discovery rate (FDR) of 1% at PSM level. The total ion current (TIC) normalization and label-free quantification was performed by Peaks Q module in PEAKS 7. The Peaks PTM module of PEAKS 7 software was used to identify sequences of glycosylation and methylation. Carbamidomethylation (C)/+57.0215 Da was set as the fixed, whereas oxidation (M)/+15.9949 Da and the following glycosylation were specified as variables: hexose modified CRKTW (+162.0528 Da), fucose modified TS (+146.0579 Da), O-GlcNac modified STN (+203.195 Da), Hex1HexNAc1 modified N (+511.1901 Da), Hex1HexNAc1NeuAc1 modified NTS (+656.2276 Da), and Hex1HexNAc1NeuAc2 modified NTS (+947.3231 Da). Also, the following methylation were specified as variables: methylation modified CDEHIKLNQRST (+14.0156 Da), dimethylation modified KNR (+28.0313 Da), K (+32.0564 Da), K (+34.0631 Da), and trimethylation modified KAR (+43.0058 Da). The MS/MS data are available through ProteomeXchange Consortium via the PRIDE partner repository with the dataset identifier PXD024997 [33].

### 2.5. MRM Method

The MRM method was performed on a 1260 Infinity II Quaternary Pump LC system (Agilent, Santa Clara, CA, USA) connected to an Agilent 6470 triple quadrupole mass spectrometer (Agilent, Santa Clara, CA, USA) in the dynamic multiple-reaction monitoring (dMRM) mode. Tryptic-digested samples were loaded onto a 50-mm-long C18 column (Phenomenex, Torrance, CA, USA) packed with 2.6-μm particles with a pore size of 100 Å and were separated using an optimized gradient in 15 min, from 5% to 15% solvent B (acetonitrile with 0.1% formic acid) at a flow rate of 0.4 mL/min and a column temperature of 40 °C. Solvent A was 0.1% formic acid in water. The mass spectrometer was operated in the dMRM mode. The samples in each batch were randomly analyzed. dMRM data was processed with Skyline 20.1.0.76 (MacCoss Lab Software, Seattle, WA, USA) and normalized by internal standards. Details of method validation are provided in “Supplementary Information”.

### 2.6. Statistical Anaylses

In targeted LC-MS/MS, the significance levels of PF4, FIBA, ITIH4, AACT, APOE, and CFAH were determined using the Student’s *t*-test. A one-way analysis of variance (ANOVA) was used to test levels among early-stage CRC, late-stage CRC, and HCs. Scheffe’s post-hoc test was applied to evaluate differences in the mean between any two groups; in addition, a post-hoc test using the Bonferroni method was applied with a 0.0167-adjusted significance level in three groups. In semi-quantification of peptides, the significance levels of PF4, ITIH4, and APOE were determined using the Student’s *t*-test. ANOVA was used to test levels among early-stage CRC, late-stage CRC, and HCs. Scheffe’s post-hoc test was applied to evaluate differences in the mean between any two groups; in addition, a post-hoc test using the Bonferroni method was applied with a 0.0167-adjusted significance level in three groups’ comparison and 0.0083-adjusted significance level in four groups comparison. We used GraphPad Prism (vers. 5.0; GraphPad Software, San Diego, CA, USA) to evaluate differences among groups and generated receiver operating characteristic (ROC) curves to evaluate the diagnostic performance of the biomarkers. The cutoff value for an ROC curve was determined by the Youden index, which represents the sum of sensitivity and [1 − specificity], and the maximum value of the Youden index is a suitable cutoff point for that curve. Pair-wise comparisons of ROC curves were assessed using MedCalc Statistical Software (vers. 15.4; MedCalc Software, Ostend, Belgium). The one-way ANOVA and power were determined using SAS (vers. 9.3; SAS Institute, Cary, NC, USA), and power estimations were calculated according to the ROC analysis. The area under the ROC curve (AUC), sensitivity, and specificity were calculated at a 95% confidence level. The significance level of all statistical tests was set to *p* < 0.05. To combine the diagnostic performance from multiple biomarkers, we incorporated four different algorithms, including logistic regression (LR), decision trees (DT), random forests (RF) and support vector machine (SVM) with 10-fold cross validation in scikit-learn (vers. 0.21.3). Parameter tuning was performed for each training and validation set on the basis of the 10-fold cross-validation. Further, the tuning process was based on the value of AUC. For RF, we used the initial tree value number of 100, which increased by 100 until reaching 500. The kernel of the model was set to gini or entropy. As for DT, we used the initial value of tree depth, which was set to 1–10 with a step of 1. The kernel of the model was set to gini or entropy. For SVM, the initial value of gamma was set to 1 × 10^−6^–1 × 10^−10^ with a step of 1e-1. The initial value of C was set to 1 × 10^−4^–1 × 10^−7^ with a 10-fold step. The kernel of the model was set to RBF. Lastly, we used the default setting in LR. To assess the predictive performance, we applied a confusion matrix to calculate the accuracy, sensitivity, specificity, and AUC.

## 3. Results

### 3.1. Discovery MS

In this study, biomarker candidates were identified using pooled samples of 20 plasma samples corresponding to CRC stage I, CRC stage II, CRC stage III, CRC stage IV, all-stage CRC, and HC samples as the discovery set (Table 1). Pooled samples were individually purified by WGA chromatography. WGA-captured plasma samples were trypsin-digested and analyzed in an LTQ-Orbitrap-Elite instrument with two replicates. Peptides corresponding to WGA-captured proteins were identified and label-free quantification was performed with PEAKS 7 software. Differentially expressed unique peptides derived from WGA-captured proteins among the early-stage CRC (stage I and stage II), late-stage CRC (stage III and stage IV), and HC groups are shown in Table S1. In this study, 55 proteins and 356 peptides were identified differentially with a false discovery rate (FDR) of 1%. The peptides were considered significantly changed if −10lgP < 13 (*p*-value < 0.05); of these, 269 peptides were increased by >1.5-fold in WGA-captured plasma from CRC groups compared to the HC group, and 4 peptides were decreased by <0.8-fold in the CRC group (Table S1). To ensure that peptide characteristics were suitable for analysis, we excluded peptides with the following signatures: (1) mis-cleavage peptides, which may lack the reproducibility in each analysis and (2) peptides containing more than 10 amino acids, which may increase the difficulty of synthesizing [34,35]. In total, 79 peptides were selected for further analysis in the pooled samples from early-stage CRC, late-stage CRC, and HC samples in the discovery set (Table 1). These selected peptides corresponding to differentially expressed WGA-captured plasma proteins were then analyzed using an Agilent 6470 instrument to examine the quality of signals on a triple quadrupole. We discovered that only two methylated peptides and four unmodified peptides satisfied a signal-to noise ratio (S/N) of >5, including ADLS*GITGAR (AACT^341–350^), HITSLEVIK (PF4^54–62^), LALDNGGLAR (ITIH4^429–438^), LGPLVEQGR (APOE^198–207^), QLEQVIAK (FIBA^202–210^), and SLGN*VIMVCR (CFAH^58–67^) (* indicates a methylated site, Table S2). Information on targeted transitions, optimized collisions, and fragmentors is summarized in Table S2.

### 3.2. Targeted LC-MS/MS

**Figure 1:**
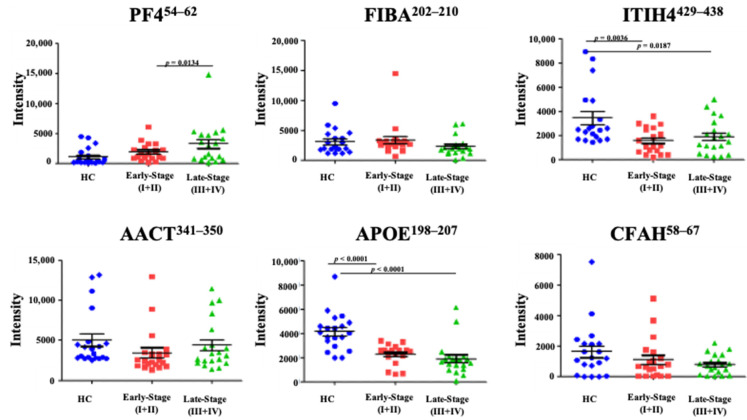
Dot plots of plasma concentrations of selected peptides corresponding to wheat germ agglutinin (WGA)-captured proteins: ADLS*GITGAR (Alhpa-1-antichymotrypsin, AACT^341–350^), HITSLEVIK (Platelet factor 4, PF4^54–62^), LALDNGGLAR (Inter-alpha-trypsin inhibitor heavy chain 4, ITIH4^429–438^), LGPLVEQGR (Apolipoprotein E, APOE^198–207^), QLEQVIAK (Fibrinogen alpha chain, FIBA^202–210^), and SLGN*VIMVCR (Complement factor H, CFAH^58–67^) in 20 paired healthy control (HC), early-stage colorectal cancer (CRC), late-stage CRC samples. The intensity was normalized with peak area of spiked Bovine serum albumin (BSA). *p* < 0.0167 was considered statistically significant.

To examine the discriminative ability of these biomarker candidates in early-stage CRC, late-stage CRC, and HC groups, we performed targeted LC-MS/MS to analyze another randomly selected 20 paired early-stage CRC, late-stage CRC, and HC plasma samples individually from the discovery set (Table 1). Plasma samples were analyzed with UPLC-MS/MS after purification with WGA chromatography and trypsin digestion. Results showed that four of six peptides corresponding to WGA-captured proteins changed in early-stage CRC compared to the HC group. The statistically significant *p* value was set to 0.0167 in a one-way analysis of variance (ANOVA). Levels of FIBA^202–210^ and PF4^54–62^ (*p* = 0.0134) increased in late-stage CRC groups compared to the HC group (Figure 1). Levels of ITIH4^429–438^ (*p* = 0.036), APOE^198–207^ (*p* < 0.0001), and CFAH^58–67^ decreased in early-stage CRC groups compared to the HC group. Among these results, PF4^429–438^ expression levels increased the most in early-stage and late-stage CRC, while APOE^198–207^ and ITIH4^429–438^ expression levels decreased the most in early-stage and late-stage CRC (Figure 1). Therefore, three peptides (PF4^54–62^, ITIH4^429–438^, and APOE^198–207^) from among the WGA-captured proteins were selected as biomarker candidates for further validation and semi-quantification with the stable isotope-labeled MRM assay.

### 3.3. Analytical Method Development

In order to ensure that our MRM assay was acceptable for use, extended stable isotope-labeled peptides and extended peptides corresponding to the three selected WGA-captured proteins (PF4^54–62^, ITIH4^429–438^, APOE^198–207^) were synthesized for semi-quantification. Details of extended peptides and extended stable isotope-labeled peptides are shown in Table S3. To validate the analytical method, the following parameters were evaluated: calibration curve, analytical specificity (selectivity), analytical sensitivity, and carryover. Details of all procedures are summarized in “Supplementary Information”. Analytical measurement ranges, including the lower limit of quantification (LLOQ) and upper limit of quantification (ULOQ), in chicken serum were 3.90 ~ 1000 ng/mL for PF4^54–62^, 1.95~250 ng/mL for ITIH4^429–438^, 1.95–250 ng/mL for APOE^192-207^, and were linear (R^2^ > 0.99) for all peptides (Table S4). The analytical specificity and sensitivity satisfied the criteria when calculated with respect to the LLOQ sample (Tables S5 and S6). The absence of carryover was confirmed by analyzing ULOQ samples, followed by a blank sample (Table S7). All of these results proved the reliability of the MRM method we developed.

### 3.4. Semi-Quantification of Peptides in Large Samples

**Figure 2:**
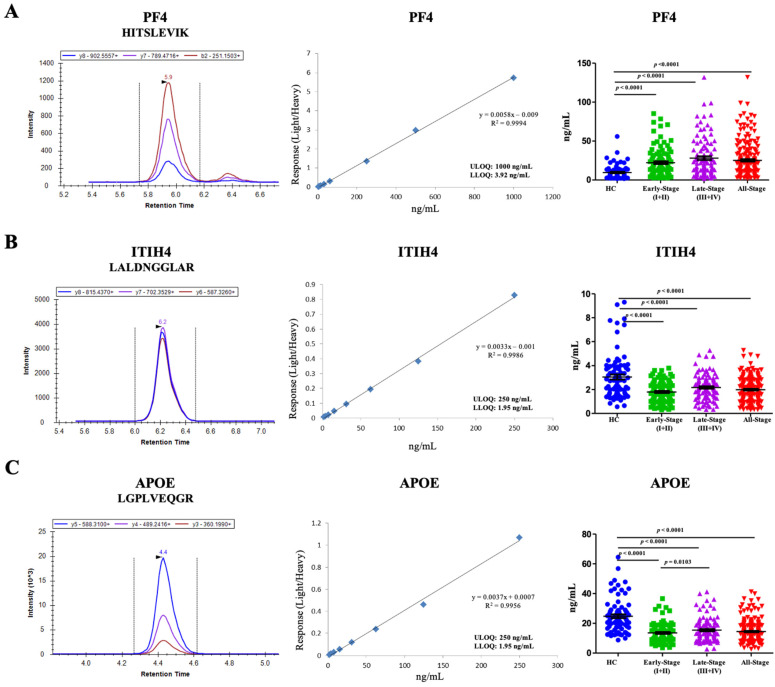
Semi-quantification of peptide factor 4 (PF4) (**A**), inter-alpha-trypsin inhibitor heavy chain H4 (ITIH4) (**B**), and apolipoprotein E (APOE) (**C**) in healthy control (HC), early-stage colorectal cancer (CRC), late-stage CRC, and all-stage CRC groups using an isotope-labeled multiple reaction monitoring (MRM) assay. Extracted ion chromatography (left panel), calibration curves (middle panel), and group comparisons of concentrations (right panel) of the three peptides are demonstrated. The mean value and standard deviation of the peptides corresponding to wheat germ agglutinin (WGA)-captured proteins from HC, early-stage CRC, late-stage CRC, and all-stage CRC groups are shown. The Student’s *t*-test was used for the pairwise comparisons of the concentration of peptides from the HC, early-stage CRC, late-stage CRC, and all-stage CRC groups. *p* < 0.0083 was considered statistically significant.

On the basis of results from targeted LC-MS/MS and analytical performance development, three peptides corresponding to PF4, ITIH4, and APOE were measured in 100 early-stage CRC, 106 late-stage CRC, and 80 HC samples (Table 1). The extracted ion chromatograms, standard curves, and group comparisons of concentrations are presented in Figure 2. The statistically significant *p* value was set to 0.0083 in a one-way analysis of variance (ANOVA). The HITSLEVIK peptide corresponding to PF4 in patients with early-stage CRC (2.65-fold, *p* < 0.0001) and late-stage CRC (2.90-fold, *p* < 0.0001) was significantly higher than that of the HC group (Figure 2A). The LALDNGGLAR peptide corresponding to ITIH4 in patients with early-stage CRC (0.68-fold, *p* < 0.0001) and late-stage CRC (0.69-fold, *p* < 0.0001) was significantly lower than that of the HC group (Figure 2B). The LGPLVEQGR peptide corresponding to APOE in patients with early-stage CRC (0.58-fold, *p* < 0.0001) and late-stage CRC (0.68-fold, *p* < 0.0001) was significantly lower than that of the HC group (Figure 2C).

### 3.5. Diagnostic Performance

**Figure 3:**
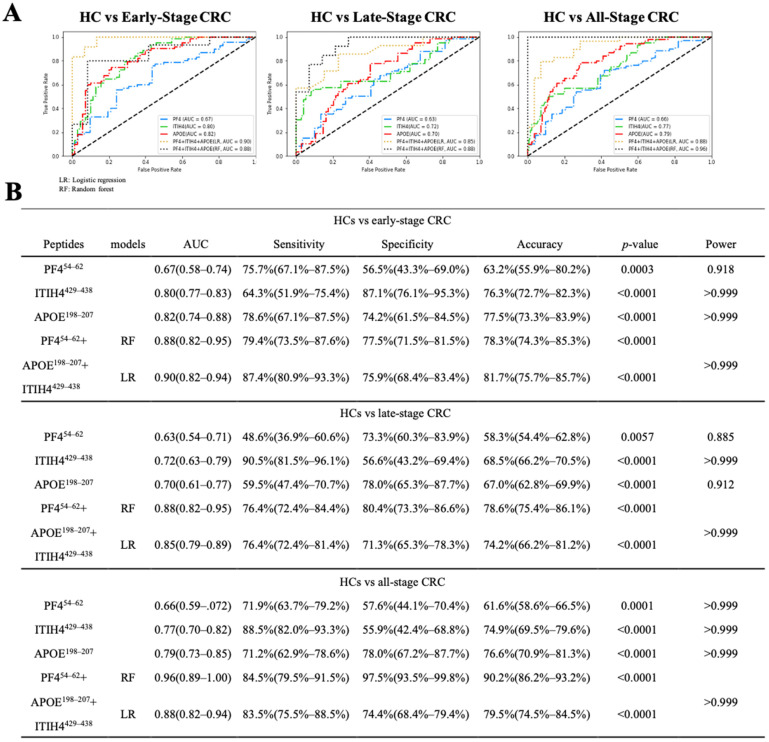
Comparison of receiver operating characteristics (ROC) curves (**A**) and predictive performance (**B**) from peptide factor 4 (PF4^54–62^), inter-alpha-trypsin inhibitor heavy chain H4 (ITIH4^429–438^), apolipoprotein E (APOE^198–207^), and combinations of the three biomarkers with logistic regression and random forest in healthy controls (HCs) versus early-stage colorectal cancer (CRC), HC versus late-stage CRC, and HC versus all-stage CRC.

We first evaluated the diagnostic performances of individual peptide biomarkers of PF4^54–62^, ITIH4^429–438^, APOE^198–207^ and determined values of the area under the receiver operating characteristics (ROC) curve (AUC) of 0.67, 0.80, and 0.82 in early-stage CRC; 0.63, 0.72, and 0.70 in late-stage CRC; and 0.66, 0.77, and 0.79 in all-stage CRC, respectively. The power estimations in this study were all above 0.885. To gain further insights into the utility of the three biomarkers, we performed several predictive models that were evaluated by the AUC, sensitivity, specificity, and accuracy. The predictive performances of decision trees, random forests, support vector machines, and logistic regressions based on combinations of the three peptide biomarkers are summarized in Table S8. Of all the models we built, the random forest (RF) and logistic regression (LR) models achieved the best predictive performances. With the combination of two peptide biomarkers in early-stage CRC detection, we observed that PF4^54–62^ + APOE^198–207^ with RF increased the AUC to 0.88 (*p* < 0.0001), the sensitivity was 81.5%, and the specificity was 82.5%. As for the combination of three peptide biomarkers in early-stage CRC detection, PF4^54–62^ + APOE^198–207^ + ITIH4^429-439^ with LR increased the AUC to 0.90 (*p* < 0.00001), the sensitivity was 87.4%, and the specificity was 75.9% (Figure 3A,B). Further, with the combination of two peptide biomarkers in late-stage CRC detection, we observed that PF4^54–62^ + APOE^198–207^ with LR increased the AUC to 0.84 (*p* < 0.0001), the sensitivity was 78.3%, and the specificity was 73.8%. As for the combination of three peptide biomarkers in late-stage CRC detection, PF4^54–62^ + APOE^198–207^ + ITIH4^429-439^ with RF increased the AUC to 0.88 (*p* < 0.00001), the sensitivity was 76.4%, and the specificity was 80.4% (Figure 3A,B). Moreover, in a combination of two peptide biomarkers in all-stage CRC detection, ITIH4^429–438^ + APOE^198–207^ combined with RF increased the AUC to 0.94 (*p* < 0.0001), the sensitivity was 80.6%, and the specificity was 96.3%. In a combination of three peptide biomarkers in all-stage CRC detection, PF4^54–62^ + APOE^198–207^ + ITIH4^429–438^ combined with RF increased the AUC to 0.96 (*p* < 0.00001), the sensitivity was 84.5%, and the specificity was 97.5% (Figure 3A,B). The combination of all three peptide biomarkers proved to be highly discriminatory for early-stage and all-stage CRC.

## 4. Discussion

To the best of our knowledge, this is the first study to combine WGA chromatography with an MRM assay and applied them in developing diagnostic biomarkers for CRC. Abundant proteins in plasma samples result in ion suppression and a matrix effect. In a previous study, WGA was used to remove abundant proteins such as albumin and enriched glycoproteins [28]. A pooling strategy by equalizing samples proved to be useful in biomarker discovery [36]. A pooling strategy can help overcome resource constraints while many individuals are analyzed; further, the variation in biological samples should be reduced and should provide increased power for detecting differences [37]. Herein, we subjected pooled CRC plasma samples and pooled HCs to WGA chromatography. After that, an LTQ elite Orbitrap mass spectrometer instrument was used to analyze the samples. In the discovery set, 55 plasma proteins and 356 plasma peptides in total were identified. We selected the peptides that received -10lgP <13 (*p*-value < 0.05) and were increased by >1.5-fold or decreased by <0.8-fold in the CRC groups for further validation. In addition to O-GlcNacylation, several PTMs that are related with cancer were also searched with Peaks PTM module, including glycosylation and methylation [38,39]. However, we found that the differentially expressed peptides were mostly unmodified and methylated. We speculated that glycosylation on peptides such as O-GlcNacylation may alter the ionization efficiency of peptides [40]. A study from Phueaouan et al. suggested O-GlcNacylation is enhanced in primary colorectal cancer tissues [41]. On a contradictory note, Krzeslak et al. showed that O-GlcNacylated protein levels were decreased in thyroid tumors [42]. Furthermore, few studies have been done to examine associated levels of glycosylated protein in blood from CRC. Thus, there is insufficient evidence to make a claim about the effect of glycosylation in cancer.

To further verify the results from the discovery set, the pooled plasma samples were purified, and differentially expressed peptides were analyzed on an Agilent 6470 triple-quadrupole mass spectrometer instrument to optimize the parameters in the LC and MS system. Also, the sensitivity (S/N > 5) and specificity (retention time within 0.1 in three transitions) were evaluated in this experiment to exclude invalid biomarker candidates. Finally, we received 6 peptides that satisfied the criteria. To further evaluate the discriminant ability of the diagnostic biomarker candidates, targeted LC-MS/MS was applied to confirm the differentially expressed peptides in the discovery set. A reference protein normalization (RFN) technique was applied in targeted LC-MS/MS. Zauber H. et al. suggested that a different species protein can be used as the reference protein to normalize and quantify without interfering with the co-analyzed sample peptides; the RFN technique is based on the addition of a protein of known concentration for normalization of sample peptide intensities [43]. Therefore, we added Bovine serum albumin (BSA) as a reference protein to 20 paired WGA-purified plasma samples to normalize the impacts from the matrix of samples. We discovered that unmodified peptides corresponding to WGA-captured PF4, ITIH4, and APOE were significantly different between CRC and HCs. Next, we attempted to develop a method to measure the glycoprotein level by targeting its unmodified peptides. The standard peptides and internal standard peptides with cleavage sites can provide insight into the cleavage process; for instance, extended stable isotope-labeled peptides were spiked into samples before tryptic digestion can compensate the digestion variability [44]. Further, studies showed that extended stable isotope-labeled peptides can be used as internal standards to account for sample processing and can provide precise and accurate results during LC-MS/MS assays [44,45]. Thus, extended peptides (as reference standards) and isotope-labeled extended peptides (as internal standards) corresponding to PF4, ITIH4, and APOE were synthesized to correct for the digestion efficiency, matrix effect, and instrument deviation. To simulate the matrix during analytical method development, an elegant study performed by Chen et al. suggested that chicken serum can be used as an alternative matrix when developing analytical methods [46]. To evaluate the performance of the analytical method, chicken serum samples were purified with WGA chromatography and spiked with exogenous peptides. Parameters including the calibration curve, sensitivity, selectivity, and carryover were evaluated in this study and proved to be acceptable for measuring by following the guideline from the U.S. Food and Drug Administration (FDA) [47]. Therefore, reliable semi-quantification of peptides corresponding to WGA-captured proteins was achieved. Finally, significantly altered peptides were semi-quantitated and evaluated in 400 plasma samples with the isotope-labeled MRM assay.

The in vitro diagnostic multivariate index assay (IVDMIA) was approved by the U.S. FDA in 2007. The score from the IVDMIA is calculated from a number of measurement values using algorithms [48]. Recently, machine learning has been combined with multiplex technologies to develop an IVDMIA; for example, Zang et. al. performed UPLC-MS/MS and machine learning methods to develop a metabolite-based IVDMIA to predict prostate cancer [49]. Hyun et al. used five biomarkers and applied algorithms, including random forest, a support vector machine, and logistic regression, to a validation cohort to predict non-small cell lung cancer [50]. Different algorithms may affect the diagnostic performance due to their characteristics. For example, LR is a traditional statistical model which is a linear model while RF is an ensemble learning method. In this study, PF4^54–62^, ITIH4^429–438^, and APOE^198–207^ were incorporated with four machine learning algorithms to predict early-stage and all-stage CRC. In early-stage prediction, we received an AUC of 0.90, a sensitivity of 87.4%, and a specificity of 75.9% with LR (Figure 3A,B). In all-stage CRC, we received an AUC of 0.96, a sensitivity of 84.5% and a specificity of 97.5% with RF (Figure 3A,B). Further, many studies have applied MS with machine learning algorithms to develop diagnostic tools in CRC diagnosis. Marin-Vincente et al. utilized THBS1 and APOC3 with a decision tree classifier and achieved an AUC of 0.83 (with a sensitivity of 90% and a specificity of 65%) in CRC diagnoses [51]. Another study carried out by Xie et al. used CELA1, CEL2A, CTRL, and TRY2 with a logistic regression and achieved an AUC of 0.90 (with a sensitivity of 86.7% and a specificity of 83.3%) in CRC diagnoses [52]. Bhardwaj et al. utilized AREG, MASP1, OPN, PON3, and TR with the least absolute shrinkage and selection operator (LASSO) and achieved an AUC of 0.86 (with a sensitivity of 83% and a specificity of 80%) in diagnosing early-stage CRC [53]. In another study from Bhardwaj et al., they combined HP, LRG1, and PON3 with LASSO and achieved an AUC of 0.83 (with a sensitivity of 67% and a specificity of 80%) in diagnosing early-stage CRC; they also utilized eight biomarkers with LASSO and achieved an AUC of 0.96 (with a sensitivity of 93% and a specificity of 80%) in diagnosing late-stage CRC [54]. In this study, PF4^54–62^, ITIH4^429–438^, and APOE^198–207^ were incorporated with four machine learning algorithms to predict early-stage and late-stage CRC. Compared to studies mentioned above, our results can provide higher sensitivity and higher specificity in diagnosing early-stage CRC and all-stage CRC with only three biomarkers. To further calculate the required sensitivity and specificity for our method to be of sufficient clinical performance for screening, Lord S. J. et al. suggested that the performance of an old test should be used as a reference to evaluate the new test [55]. Therefore, we compared our method with FOBT which is the gold standard for CRC screening. A meta-analysis from Ramdzan A. R. et al. reported that the sensitivity and specificity of FOBT are 31% and 87%, respectively [56]. In our study, PF4^54–62^, ITIH4^429–438^, and APOE^198–207^ combined by RF can provide the sensitivity 84.5 % and specificity 97.5% on diagnosis all-stage CRC, which is better than FOBT.

The potential biomarkers we discovered in this study are related to highly abundant plasma proteins; for these, it is easy to detect the alteration in plasma. However, our method is to measure the unmodified peptides derived from glycosylated protein which still provide the specificity for diagnostic test. PF4 is known as an endocrine factor and is preserved in α-granules of megakaryocytes and mature platelets [57]. Although PF4 is not a glycoprotein, studies indicated that PF4 may bind to glycoprotein [58,59]. Furthermore, Muramatsu T. et al. suggested a glycoprotein binding protein can be captured by affinity chromatography, such as lectin chromatography [60]. We identified PF4 in WGA-binding proteins from CRC patients and HCs in a discovery study and further validated PF4 in the large samples. A study by Pucci et al. suggested that higher levels of PF4 in tumors can cause a poor survival rate in patients; additionally, they found that overproduction of PF4 can accelerate de novo adenocarcinogenesis and suggested that platelets can modulate the tumor microenvironment by releasing PF4 [61]. Zhang et al. discovered that PF4 can induce CRC recurrence in patients who received chemotherapy by suppressing antitumor immunity [62]. A study by Peterson et al. found that PF4 in platelets from CRC patients was significantly higher compared to levels in HCs; using a logistic regression, they achieved a sensitivity of 82.8% and a specificity of 79.4% in diagnosing CRC [63]. In our study, we observed that PF4 was significantly increased in early-stage CRC (2.65-fold) and late-stage CRC (2.90-fold) compared to healthy controls, which is consistent with previous studies. Altogether, our results and the literature indicate that PF4 is associated with CRC progression.

ITIH4 is secreted by the liver into the circulation and is related to inflammation. Further, ITIH4 was found to mediate tumorigenesis, invasion, and metastasis of solid tumors [64,65]. Huang et al. discovered that a decreasing level of ITIH4 promoted the invasion and metastasis of ovarian cancer cells; moreover, they also found that ITIH4 was downregulated in tissues from patients with ovarian cancer compared to HCs [66]. An elegant study of liver cancer by Lee et al. suggested that increasing levels of ITIH4 can suppress tumor invasion, whereas decreasing levels of ITIH4 can promote tumor metastasis [67]. A retrospective study by Hamm et al. showed that the gene expression level of ITIH4 decreased in tissues derived from patients with CRC [68]. Further, detection of ITIH4 by an enzyme-linked immunosorbent assay was applied to patients with CRC and achieved a sensitivity of 78.2% and a specificity of 76.3% [69]. In our study, a decreasing level of ITIH4 was observed in early-stage CRC (0.68-fold) and late-stage CRC (0.69-fold), which is consistent with previous studies.

APOE, a glycoprotein that is associates with triglyceride-rich lipoproteins, were predominantly synthesized in the liver; further, APOE mediates the clearance of triglyceride and lipoprotein remnants [70]. While it is unclear exactly how APOE is involved in cancer development, it may affect the metabolism of cancer cells by regulating lipid homeostasis [71]. Further, Pencheva et al. suggested that APOE secreted from melanoma cells can bind to APOE receptors on melanoma cells and endothelial cells to inhibit invasiveness and clustering [72]. A study by Buss et al. showed that APOE^−/−^ breast cancer tumors grew faster than controls in mouse experiments, which indicates that APOE may inhibit cancer development [73]. Moreover, EI-Bahrawy et al. suggested that APOE plays an important role in metabolism in the colon; the absence of APOE can stimulate the expression of cyclooxygenase (COX)-2 by increasing oxidized low-density lipoprotein (Ox-LDL) and tumor necrosis factor (TNF)-α, which induces inflammation and results in colon disease [74]. Also, APOE was observed to have decreased in lung cancer tissues [75]. Decreases in APOE in patients with early-stage CRC (0.58-fold) and late-stage CRC (0.68-fold) in this study indicated that APOE may play roles in suppressing tumor activity, which is consistent with previous studies.

## 5. Conclusions

In the present study, we discovered that PF4^54–62^ was greatly increased while ITIH4^429–438^ and APOE^198–207^ were significantly decreased in CRC patients. Moreover, we combined PF4^54–62^, APOE^198–207^, and ITIH4^429–438^ with RF to predict all-stage CRC and received an AUC of 0.96, a sensitivity of 84.5% and a specificity of 97.5%. In conclusion, our study demonstrated that WGA-captured plasma PF4^54–62^, ITIH4^429–438^, and APOE^198–207^ levels in combination may serve as highly effective early diagnostic biomarkers for patients with CRC.

## Figures and Tables

**Table 1:** Demographic information of patients with colorectal cancer (CRC) and healthy controls (HCs).

Varible	Discovery Set					Validation Set				
group	HC	early-stage CRC		late-stage CRC		HC	early-stage CRC		late-stage CRC	
number of samples	*n* = 40	*n* = 40		*n* = 40		*n* = 80	*n* = 100		*n* = 106	
		stage I	stage II	stage III	stage IV		stage I	stage II	stage III	stage IV
		*n* =20	*n* =20	*n* =20	*n* =20		*n* = 47	*n* =53	*n* =50	*n* =56
male: female ratio	24:16	24:16		24:16		46:34	71:29		66:40	
mean age years ± SD	57.65 ± 3.48	52.5 ± 6.87		51.25 ± 4.85		39.43 ± 11.14	70.84 ± 10.7		67.33 ± 10.06	

## Data Availability

The mass spectrometry proteomics data have been deposited to ProteomeXchange Consortium via the PRIDE [33] partner repository with the dataset identifier PXD024997.

## Supplementary Materials

The following are available online at https://www.mdpi.com/article/10.3390/cancers13092190/s1, Table S1. Differentially expressed peptides derived from wheat germ agglutinin (WGA)-captured proteins with nanoLC-MS/MS from healthy control groups compared to patients at various colorectal cancer (CRC) stages, Table S2. Multiple-reaction monitoring (MRM) transitions and mass parameters of selected peptides, Table S3. Information of the synthetic extended peptides and their internal standards, Table S4. Results of the calibration curve analysis, Table S5. Results of the specificity analysis. Table S6. Results of the sensitivity analysis, Table S7. Results of the carryover analysis, Table S8. Predictive performance and power analysis using peptide factor 4 (PF4), inter-alpha-trypsin inhibitor heavy chain H4 (ITIH4), apolipoprotein E (APOE), and combinations of the three biomarkers, File S1: The supplementary information for analytical method development.
