# Supplementary material for: Clinical Assay for the Early Detection of Colorectal Cancer Using Mass Spectrometric Wheat Germ Agglutinin Multiple Reaction Monitoring
Source: Cancers (Basel). 2021 May 2;13(9):2190. doi: 10.3390/cancers13092190 (PMC8124906; doi:10.3390/cancers13092190)
Supplement: Supplementary file 1 [file cancers-13-02190-s001.zip › 1 Table S2 Multiple-reaction monitoring (MRM) transitions and mass parameters of selected peptides.pdf]

Table S2 Multiple-reaction monitoring (MRM) transitions and mass parameters of selected peptides

Protein Name	Peptide sequence	Time(min)	MRM(m/z)	CE (eV)	Fragmentor
P01011|AACT	ADLS[+14.01565]GITGAR	6.22	487.7> 675.3; 300.1; 187.0	9.3	130
P02776|PF4	HITSLEVIK	5.9	520.3> 902.5; 789.4; 251.1	20.1	130
P02649|APOE	LGPLVEQGR	4.4	484.7> 588.3; 489.2; 360.1	22	130
Q14624|ITIH4	LALDNGGLAR	6.2	500.2>815.4; 702.3; 587.3	16.5	130
P08603|CFAH	SLGN[+14.01]VIMVC[+57.02]R	4.74	581.8> 962.4; 678.3; 565.2	14.1	130
P02671|FIBA	QLEQVIAK	3.1	464.7> 430.3; 331.2; 218.1	14.1	130
