# Supplementary material for: Clinical Assay for the Early Detection of Colorectal Cancer Using Mass Spectrometric Wheat Germ Agglutinin Multiple Reaction Monitoring
Source: Cancers (Basel). 2021 May 2;13(9):2190. doi: 10.3390/cancers13092190 (PMC8124906; doi:10.3390/cancers13092190)
Supplement: Supplementary file 1 [file cancers-13-02190-s001.zip › 1 Table S3 Information of synthetic extended peptides.pdf]

Table S3. Information of the synthetic extended peptides and their internal standards

Protein	Peptide	Position in protein[a]	Isotope labeled	Precursor ion (m/z); charge	Product ion (m/z); charge	Product ion type	time (min)	CE (eV)	Extended peptide	Position in protein[a]
PF4	HITSLEVIK	54-62	unlabeled	520.3;2	902.5;1	y8	5.9	20.1	VRPRHITSLE<br>VIKAGPH	50-66
					789.4;1	y7				
					251.1;1	b2				
PF4	HITS**L**E**V**IK	54-62	labeled	526.8;2	915.5;1	y8	5.9	20.1	VRPRHITS**L**E<br>**V**IKAGPH	50-66
					802.5;1	y7				
					251.1;1	b2				
ITIH4	LALDNGGLAR	429-438	unlabeled	500.2;2	815.4;1	y8	6.2	16.5	FLEKLALDN<br>GGLARRIHE	425-442
					702.3;1	y7				
					587.3;1	y6				
ITIH4	LA**L**DNGGL**A**R	429-438	labeled	505.7;2	826.4;1	y8	6.2	16.5	FLEKLA**L**DN<br>GGL**A**RRIHE	425-442
					706.3;1	y7				
					591.3;1	y6				

APOE	LGPLVEQGR	198-207	unlabeled	484.7;2	588.3;1	y5	4.4	22	IRERLGPLVE QGRVRAA	194-211
					489.2;1	y4				
					360.1;1	y3				
APOE	LGP**LV**EQGR	198-207	labeled	491.2;2	594.3;1	y5	4.4	22	IRERLGP**LV**E QGRVRAA	194-211
					489.2;1	y4				
					360.1;1	y3				
