# Supplementary material for: Clinical Assay for the Early Detection of Colorectal Cancer Using Mass Spectrometric Wheat Germ Agglutinin Multiple Reaction Monitoring
Source: Cancers (Basel). 2021 May 2;13(9):2190. doi: 10.3390/cancers13092190 (PMC8124906; doi:10.3390/cancers13092190)
Supplement: Supplementary file 1 [file cancers-13-02190-s001.zip › 1 Table S4 Results of the calibration curve analysis.pdf]

Table S4 Results of the calibration curve analysis

Peptide	Replicates	Measurements	Calibrator 1	Calibrator 2	Calibrator 3	Calibrator 4	Calibrator 5	Calibrator 6	Calibrator 7	Calibrator 8
HITSLEVIK		Expected conc. (ng/mL)	3.906	7.812	15.625	31.250	62.000	250.000	500.000	1000.000
	Replicate 1	PAR	0.018	0.035	0.069	0.146	0.310	1.350	2.968	5.729
	Replicate 2	PAR	0.018	0.040	0.076	0.179	0.312	1.525	2.839	6.435
	Replicate 3	PAR	0.017	0.041	0.076	0.181	0.360	1.596	3.277	6.598
		SD	0.000	0.002	0.033	0.016	0.023	0.103	0.183	0.377
		CV (%)	2.668	6.787	4.479	9.514	7.061	6.936	6.069	6.030
LALDNGGLAR		Expected conc. (ng/mL)	1.953	3.906	7.812	15.625	31.25	62.500	125.000	250.000
	Replicate 1	PAR	0.006	0.011	0.021	0.048	0.094	0.194	0.381	0.827
	Replicate 2	PAR	0.005	0.011	0.025	0.046	0.091	0.180	0.453	0.773
	Replicate 3	PAR	0.007	0.014	0.029	0.060	0.104	0.227	0.367	0.945
		SD	0.000	0.001	0.003	0.006	0.005	0.019	0.037	0.007
		CV (%)	13.608	11.785	13.063	12.043	5.769	9.835	9.411	8.466

LGPLVEQGR		Expected conc. (ng/mL)	1.953	3.906	7.812	15.625	31.25	62.500	125.000	250.000
	Replicate 1	PAR	0.007	0.014	0.029	0.054	0.117	0.239	0.460	1.038
	Replicate 2	PAR	0.009	0.017	0.032	0.064	0.130	0.238	0.499	1.036
	Replicate 3	PAR	0.009	0.017	0.033	0.068	0.120	0.254	0.485	1.061
		SD	0.000	0.000	0.001	0.001	0.005	0.007	0.016	0.011
		CV (%)	11.313	8.838	5.424	9.496	4.543	3.003	3.351	1.085

PAR: Peak area ratio
