# Supplementary material for: Clinical Assay for the Early Detection of Colorectal Cancer Using Mass Spectrometric Wheat Germ Agglutinin Multiple Reaction Monitoring
Source: Cancers (Basel). 2021 May 2;13(9):2190. doi: 10.3390/cancers13092190 (PMC8124906; doi:10.3390/cancers13092190)
Supplement: Supplementary file 1 [file cancers-13-02190-s001.zip › 1 Table S5 Results of the specificity analysis.pdf]

Table S5. Results of the specificity analysis

Peptide	Matrix	Peptide type	Peak area of blank sample	Peak area of LLOQ (calibrator 1)	Interference (%)[a]
**HITSLEVIK**		unlabeled			
	Replicate 1		10.250	137.727	7.442
	Replicate 2		7.146	328.214	2.177
	Replicate 3		5.388	224.401	2.401
		labeled			
	Replicate 1		13.945	5980.025	0.233
	Replicate 2		16.216	5578.894	0.290
	Replicate 3		3.894	2201.981	0.176
**LALDNGGLAR**		unlabeled			
	Replicate 1		0.1558	167.612	0.092
	Replicate 2		0.8486	533.889	0.158
	Replicate 3		0.5584	259.453	0.215
		labeled			
	Replicate 1		22.595	25106.303	0.089
	Replicate 2		8.794	98652.190	0.008
	Replicate 3		5.689	30260.840	0.018
**LGPLVEQGR**		unlabeled			
	Replicate 1		0.993	150.346	0.660
	Replicate 2		0.858	409.362	0.209
	Replicate 3		1.924	238.291	0.807
		labeled			
	Replicate 1		11.391	21344.238	0.053
	Replicate 2		13.505	44937.280	0.030

Replicate 3	11.439	26055.170	0.043

[a]Interference was calculated by dividing the peak area of the blank sample by that of the LLOQ and multiplying the result by 100.
