# Supplementary material for: Clinical Assay for the Early Detection of Colorectal Cancer Using Mass Spectrometric Wheat Germ Agglutinin Multiple Reaction Monitoring
Source: Cancers (Basel). 2021 May 2;13(9):2190. doi: 10.3390/cancers13092190 (PMC8124906; doi:10.3390/cancers13092190)
Supplement: Supplementary file 1 [file cancers-13-02190-s001.zip › 1 Table S6 Results of the sensitivity analysis.pdf]

Table S6. Results of the sensitivity analysis

Peptide	Replicates	Zero sample (PAR)	Calibrator 1(PAR)	Signal-to-noise (S/N)[a]
HITSLEVIK				
	Replicate 1	0.002	0.018	7.704
	Replicate 2	0.003	0.018	5.072
	Replicate 3	0.002	0.017	7.897
LALDNGGLAR				
	Replicate 1	0.001	0.006	6.67
	Replicate 2	0.000	0.005	13.95
	Replicate 3	0.001	0.008	8.56
LGPLVEQGR				
	Replicate 1	0.001	0.007	5.614
	Replicate 2	0.001	0.009	5.126
	Replicate 3	0.001	0.009	5.805

PAR: peak area ratio.

[a] Signal-noise-ratio was calculated by dividing the peak area ratio of LLOQ by the zero sample.
