# Supplementary material for: Clinical Assay for the Early Detection of Colorectal Cancer Using Mass Spectrometric Wheat Germ Agglutinin Multiple Reaction Monitoring
Source: Cancers (Basel). 2021 May 2;13(9):2190. doi: 10.3390/cancers13092190 (PMC8124906; doi:10.3390/cancers13092190)
Supplement: Supplementary file 1 [file cancers-13-02190-s001.zip › 1 Table S7 Results of the carryover analysis.pdf]

Table S7. Results of the carryover analysis

Peptide	Replicates	Peptide type	Peak area of blank sample	Peak area of LLOQ (calibrator 1)	Carryover (%)[a]
**HITSLEVIK**		unlabeled			
	Replicate 1		3.616	137.727	2.625
	Replicate 2		11.510	328.214	3.506
	Replicate 3		7.924	224.401	3.531
		labeled			
	Replicate 1		16.556	5980.025	0.276
	Replicate 2		14.203	5578.894	0.254
	Replicate 3		11.200	2201.981	0.508
**LALDNGGLAR**		unlabeled			
	Replicate 1		0.371	167.612	0.221
	Replicate 2		2.616	533.889	0.489
	Replicate 3		4.693	259.453	1.808
		labeled			
	Replicate 1		11.75	25106.303	0.046
	Replicate 2		16.38	98652.190	0.016
	Replicate 3		8.858	30260.840	0.029
**LGPLVEQGR**		unlabeled			
	Replicate 1		3.660	150.346	2.434
	Replicate 2		10.481	409.362	2.560
	Replicate 3		6.816	238.291	2.860
		labeled			
	Replicate 1		4.776	21344.238	0.022
	Replicate 2		13.092	44937.280	0.029
	Replicate 3		4.041	26055.170	0.015

[a] Carryover was calculated by dividing the peak area of the blank sample by that of the LLOQ and multiplying the result by 100.
