# Supplementary material for: Clinical Assay for the Early Detection of Colorectal Cancer Using Mass Spectrometric Wheat Germ Agglutinin Multiple Reaction Monitoring
Source: Cancers (Basel). 2021 May 2;13(9):2190. doi: 10.3390/cancers13092190 (PMC8124906; doi:10.3390/cancers13092190)
Supplement: Supplementary file 1 [file cancers-13-02190-s001.zip › 1 Table S8 Predictive performance.pdf]

Table. S8 Health controls vs early-stage colorectal cancer

Peptides		AUC	Sensitivity	Specificity	Accuracy	*p*-value	Power
PF4$^{54-62}$		0.67(0.58-0.74)	75.7%(67.1%-87.5%)	56.5%(43.3%-69.0%)	63.2%(55.9%-80.2%)	0.0003	0.918
ITIH4$^{429-438}$		0.80(0.77-0.83)	64.3%(51.9%-75.4%)	87.1%(76.1%-95.3%)	76.3%(72.7%-82.3%)	<0.0001	>0.999
APOE$^{198-207}$		0.82(0.74-0.88)	78.6%(67.1%-87.5%)	74.2%(61.5%-84.5%)	77.5%(73.3%-83.9%)	<0.0001	>0.999
PF4$^{54-62}$+ ITIH4$^{429-438}$	DT	0.72(0.65-0.79)	61.3%(55.4%-67.2%)	76.3%(73.3%-80.3%)	67.8%(60.8%-74.8%)	<0.0001	>0.999
	RF	0.63(0.56-0.69)	66.5%(60.8%-70.3%)	60.8%(52.6%-66.4%)	63.3%(57.3%-68.3%)	<0.0001	
	SVM	0.74(0.68-0.80)	70.4%(63.1%-73.9%)	67.5%(63.5%-72.5%)	68.9%(64.9%-72.9%)	<0.0001	
	LR	0.83(0.76-0.87)	76.8%(73.6%-83.2%)	65.4%(58.4%-71.8%)	71.1%(64.1%-74.1%)	<0.0001	
PF4$^{54-62}$+ APOE$^{198-207}$	DT	0.83(0.76-0.90)	69.3%(66.4%-75.4%)	85.7%(77.3%-92.4%)	76.1%(72.1%-84.1%)	<0.0001	>0.999
	RF	0.88(0.81-0.95)	81.5%(74.9%-88.3%)	82.5%(75.5%-89.5%)	81.7%(75.7%-89.7%)	<0.0001	
	SVM	0.82(0.74-0.87)	70.3%(62.5%-76.3%)	72.5%(67.5%-79.5%)	71.1%(68.1%-77.1%)	<0.0001	
	LR	0.88(0.83-0.95)	87.8%(80.2%-91.5%)	76.3%(71.3%-79.3%)	82.2%(74.2%-88.2%)	<0.0001	
ITIH4$^{429-438}$ + APOE$^{198-207}$	DT	0.77(0.72-0.83)	87.5%(79.1%-90.4%)	70.7%(64.6%-77.8%)	79.4%(75.4%-83.4%)	<0.0001	>0.999
	RF	0.84(0.77-0.90)	82.1%(74.9%-86.7%)	75.4%(67.1%-83.8%)	78.9%(70.9%-83.9%)	<0.0001	
	SVM	0.74(0.68-0.80)	78.1%(70.1%-84.1%)	64.3%(61.3%-72.3%)	71.7%(63.7%-79.7%)	<0.0001	
	LR	0.85(0.79-0.89)	87.5%(81.7%-95.9%)	67.5%(63.5%-72.5%)	78.3%(70.3%-86.3%)	<0.0001	
PF4$^{54-62}$+ APOE$^{198-207}$+ ITIH4$^{429-438}$	DT	0.84(0.78-0.90)	74.6%(70.6%-78.4%)	80.5%(73.9%-83.6%)	76.7%(71.7%-82.7%)	<0.0001	>0.999
	RF	0.88(0.82-0.95)	79.4%(73.5%-87.6%)	77.5%(71.5%-81.5%)	78.3%(74.3%-85.3%)	<0.0001	
	SVM	0.80(0.73-0.85)	71.4%(65.4%-77.4%)	80.5%(72.5%-88.5%)	75.6%(68.6%-81.6%)	<0.0001	
	LR	0.90(0.82-0.94)	87.4%(80.9%-93.3%)	75.9%(68.4%-83.4%)	81.7%(75.7%-85.7%)	<0.0001	

Table. S8 Health controls vs late-stage colorectal cancer

Peptides		AUC	Sensitivity	Specificity	Accuracy	*p*-value	Power
$PF4^{54-62}$		0.63(0.54-0.71)	48.6%(36.9%-60.6%)	73.3%(60.3%-83.9%)	58.3%(54.4%-62.8%)	0.0057	0.885
$ITIH4^{429-438}$		0.72(0.63-0.79)	90.5%(81.5%-96.1%)	56.6%(43.2%-69.4%)	68.5%(66.2%-70.5%)	<0.0001	>0.999
$APOE^{198-207}$		0.70(0.61-0.77)	59.5%(47.4%-70.7%)	78.0%(65.3%-87.7%)	67.0%(62.8%-69.9%)	<0.0001	0.912
	DT	0.71(0.65-0.76)	67.9%(61.9%-73.9%)	71.3%(65.3%-78.3%)	69.4%(65.4%-77.4%)	<0.0001	
$PF4^{54-62}$+	RF	0.79(0.72-0.84)	73.6%(69.6%-77.6%)	68.8%(62.8%-75.8%)	71.5%(65.5%-76.5%)	<0.0001	0.985
$ITIH4^{429-438}$	SVM	0.65(0.60-0.71)	66.7%(61.7%-72.7%)	62.5%(55.5%-70.5%)	64.8%(61.8%-69.8%)	0.0043	
	LR	0.80(0.73-0.86)	71.7%(64.7%-76.7%)	66.3%(63.3%-72.3%)	69.4%(65.4%-75.4%)	<0.0001	
	DT	0.76(0.68-0.81)	65.1%(59.1%-72.1%)	90.4%(85.1%-96%)	75.8%(71.8%-81.8%)	<0.0001	
$PF4^{54-62}$+	RF	0.82(0.77-0.88)	76.4%(71.4%-81.4%)	72.5%(64.5%-80.5%)	74.7%(67.7%-77.7%)	<0.0001	0.985
$APOE^{198-207}$	SVM	0.81(0.75-0.88)	72.9%(69.9%-76.9%)	81.3%(75.6%-87.5%)	76.7%(72.7%-83.7%)	0.0001	
	LR	0.84(0.78-0.89)	78.3%(73.3%-83.3%)	73.8%(66.8%-80.8%)	76.3%(69.3%-82.3%)	0.0001	
	DT	0.74(0.69-0.80)	83.1%(80.4%-91.1%)	46.3%(38.3%-52.3%)	67.2%(61.2%-74.2%)	<0.0001	
$ITIH4^{429-438}$+	RF	0.77(0.71-0.83)	75.5%(70.5%-78.5%)	67.5%(60.5%-72.5%)	72.4%(67.2%-79.4%)	<0.0001	>0.999
$APOE^{198-207}$	SVM	0.71(0.64-0.77)	74.1%(66.1%-80.1%)	51.4%(48.4%-57.4%)	65.7%(57.7%-73.6%)	<0.0001	
	LR	0.76(0.71-0.83)	84.5%(80.7%-87.6%)	56.3%(52.3%-64.3%)	72.4%(69.6%-77.6%)	<0.0001	
$PF4^{54-62}$	DT	0.78(0.72-0.85)	71.7%(67.7%-77.7%)	73.8%(70.8%-78.8%)	72.6%(67.6%-75.6%)	<0.0001	
+ $APOE^{198-207}$+	RF	0.88(0.82-0.95)	76.4%(72.4%-84.4%)	80.4%(73.3%-86.6%)	78.6%(75.4%-86.1%)	<0.0001	>0.999
$ITIH4^{429-438}$	SVM	0.80(0.75-0.87)	72.7%(67.7%-80.7%)	76.6%(73.6%-83.6%)	74.3%(67.3%-82.3%)	<0.0001	
	LR	0.85(0.79-0.89)	76.4%(72.4%-81.4%)	71.3%(65.3%-78.3%)	74.2%(66.2%-81.2%)	<0.0001	

Table. S8 Health controls vs all-stage colorectal cancer

Peptides		AUC	Sensitivity	Specificity	Accuracy	*p*-value	Power
PF4$^{54-62}$		0.66(0.59-.072)	71.9%(63.7%-79.2%)	57.6%(44.1%-70.4%)	61.6%(58.6%-66.5%)	0.0001	>0.999
ITIH4$^{429-438}$		0.77(0.70-0.82)	88.5%(82.0%-93.3%)	55.9%(42.4%-68.8%)	74.9%(69.5%-79.6%)	<0.0001	>0.999
APOE$^{198-207}$		0.79(0.73-0.85)	71.2%(62.9%-78.6%)	78.0%(67.2%-87.7%)	76.6%(70.9%-81.3%)	<0.0001	>0.999
PF4$^{54-62}$+ ITIH4$^{429-438}$	DT	0.72(0.64-0.77)	59.2%(51.2%-67.2%)	83.8%(77.8%-89.8%)	69.9%(66.9%-76.9%)	<0.0001	>0.999
	RF	0.89(0.83-0.95)	77.2%(71.2%-80.2%)	91.3%(88.3%-95.3%)	83.3%(77.3%-87.3%)	<0.0001	
	SVM	0.75(0.68-0.81)	71.5%(65.5%-75.5%)	88.4%(84.6%-93.5%)	74.3%(71.3%-80.3%)	<0.0001	
	LR	0.81(0.76-0.87)	75.2%(71.2%-80.2%)	64.4%(61.4%-70.4%)	70.5%(62.5%-73.5%)	<0.0001	
PF4$^{54-62}$+ APOE$^{198-207}$	DT	0.83(0.76-0.87)	79.6%(72.6%-86.6%)	76.9%(68.9%-83.9%)	78.4%(74.4%-84.4%)	<0.0001	>0.999
	RF	0.93(0.86-0.99)	80.1%(76.1%-86.1%)	96.3%(92.3%-99.3%)	87.2%(81.2%-92.2%)	<0.0001	
	SVM	0.82(0.77-0.87)	74.5%(68.5%-79.5%)	71.4%(68.4%-77.4%)	78.8%(74.8%-82.8%)	<0.0001	
	LR	0.87(0.79-0.93)	82.4%(74.8%-89%)	78.8%(70.8%-84.8%)	80.6%(76.6%-87.6%)	<0.0001	
ITIH4$^{429-438}$+ APOE$^{198-207}$	DT	0.83(0.75-0.88)	88.3%(81.3%-94.3%)	64.4%(58.4%-69.4%)	77.9%(73.9%-80.9%)	<0.0001	>0.999
	RF	0.94(0.89-1.00)	80.6%(75.6%-88.6%)	96.3%(93.3%-98.7%)	87.4%(82.4%-95.4%)	<0.0001	
	SVM	0.81(0.74-0.88)	78.7%(73.7%-82.7%)	70.1%(62.1%-77.1%)	74.5%(71.5%-82.5%)	<0.0001	
	LR	0.81(0.75-0.87)	84.6%(78.6%-92%)	64.4%(58.4%-68.4%)	75.4%(71.4%-83.4%)	<0.0001	
PF4$^{54-62}$ + APOE$^{198-207}$+ ITIH4$^{429-438}$	DT	0.88(0.83-0.93)	85.4%(80.4%-90.4%)	78.1%(72.1%-86.1%)	82.2%(76.2%-90.2%)	<0.0001	>0.999
	RF	0.96(0.89-1.00)	84.5%(79.5%-91.5%)	97.5%(93.5%-99.8%)	90.2%(86.2%-93.2%)	<0.0001	
	SVM	0.85(0.79-0.90)	84.1%(81.1%-92.1%)	74.8%(71.8%-81.8%)	81.4%(76.4%-84.4%)	<0.0001	
	LR	0.88(0.82-0.94)	83.5%(75.5%-88.5%)	74.4%(68.4%-79.4%)	79.5%(74.5%-84.5%)	<0.0001	
