# Supplementary material for: Clinical Assay for the Early Detection of Colorectal Cancer Using Mass Spectrometric Wheat Germ Agglutinin Multiple Reaction Monitoring
Source: Cancers (Basel). 2021 May 2;13(9):2190. doi: 10.3390/cancers13092190 (PMC8124906; doi:10.3390/cancers13092190)
Supplement: Supplementary file 1 [file cancers-13-02190-s001.zip › File S1.pdf]

Supplementary information

Analytical method development

Calibration curve

Beause of the presence of peptides in patients' plasma samples, it was not possible to provide the lower limit of quantification (LLOQ) when using patents' plasma as a matrix. Therefore, we used chicken serum as the matrix to generate a calibration curve to accurately and reproductibly calculate the LLOQ by adding exogenous extended synthetic peptides. The calibration curve was constructed by spiking equal amounts of extended isotope-labeled peptides with varying amounts of extended synthetic peptides at eight concentrations. Blank samples (matrix without an internal standard) and zero samples (matrix with the internal standard) were analyzed. All calibrators were prepared daily and analyzed in two replicates per day over 3 days. The limit of detection (LOD) and limit of quantification (LOQ) were determined by mean values of zero samples plus 3 and 10 times the standard deviation according to the International Conference on Harmonisation of Technical Requirements for Registration of Pharmaceuticals for Human Use (ICH).
To determine the LLOQ, spiked samples at five concentrations (0.001~5 ng/mL) were analyzed in 3 replicates over 3 day. The lowest concentration that met the precision (coefficient of variation (CV) of <20%), accuracy (85%~120%), and signal-to-noise (S/N >5) criteria was selected as the LLOQ. The upper limit of quantification (ULOQ) was defined as the highest concentration in the calibration curve that met the precision (CV of <20%) and accuracy criteria (85%~120%). A weighted linear regression model was fitted.

Analytical specificity (selectivity or interference)

Analytical specificity was calculated by comparing concentrations of peptides and isotope-labeled peptides in blank samples with LLOQ samples. The interference of peptides and isotope-labeled peptides was calculated as the percentage of the peak area of peptides and isotope-labeled peptides from blank samples with repect to the peak area of peptides and isotope-labeled peptides from the LLOQ. All samples were analyzed and calculated in 3 replicates over 3 days.

Analytical sensitivity

The LLOQ was defined as the lowest concentration on the calibration curve. To calculate the S/N, the signal and noise were presented by the peak area ratios of calibrator 1 and the zero sample in the matrix. The LLOQ samples and zero samples were prepared daily and analyzed in 3 replicates over 3 days.

Carryover

Carryover was assessed by analyzing blank samples after the ULOQ. Blank samples were prepared daily and analyzed in 3 replicates over 3 days.
